# The critical Barkhausen avalanches in thin random-field ferromagnets with an open boundary

**DOI:** 10.1038/s41598-019-42802-w

**Published:** 2019-04-19

**Authors:** Bosiljka Tadić, Svetislav Mijatović, Sanja Janićević, Djordje Spasojević, Geoff J. Rodgers

**Affiliations:** 1 Department for Theoretical Physics, Jožef Stefan Institute, P.O. Box 3000, SI-1001 Ljubljana, Slovenia; 2 Complexity Science Hub, Vienna, Austria; 3 Faculty of Physics, University of Belgrade, POB 368, 11001 Belgrade, Serbia; 4 Brunel University London, Uxbridge Middlesex, UB8 3PH UK

**Keywords:** Computational science, Statistical physics, thermodynamics and nonlinear dynamics

## Abstract

The interplay between the critical fluctuations and the sample geometry is investigated numerically using thin random-field ferromagnets exhibiting the field-driven magnetisation reversal on the hysteresis loop. The system is studied along the theoretical critical line in the plane of random-field disorder and thickness. The thickness is varied to consider samples of various geometry between a two-dimensional plane and a complete three-dimensional lattice with an open boundary in the direction of the growing thickness. We perform a multi-fractal analysis of the Barkhausen noise signals and scaling of the critical avalanches of the domain wall motion. Our results reveal that, for sufficiently small thickness, the sample geometry profoundly affects the dynamics by modifying the spectral segments that represent small fluctuations and promoting the time-scale dependent multi-fractality. Meanwhile, the avalanche distributions display two distinct power-law regions, in contrast to those in the two-dimensional limit, and the average avalanche shapes are asymmetric. With increasing thickness, the scaling characteristics and the multi-fractal spectrum in thicker samples gradually approach the hysteresis loop criticality in three-dimensional systems. Thin ferromagnetic films are growing in importance technologically, and our results illustrate some new features of the domain wall dynamics induced by magnetisation reversal in these systems.

## Introduction

Disordered ferromagnets are well-known memory materials and new classes of memory devices are increasingly making use of controlled motion of the domain walls (DW) in thin ferromagnetic films and nanowires^1–3^. The underlying magnetisation-reversal processes in these disordered ferromagnetic materials typically exhibit domain nucleation and domain-wall propagation under slow driving by the external field^4,5^. Hence, there is an increased interest in the experimental investigations of the Barkhausen noise (BHN) accompanying the magnetisation reversal along the hysteresis loop in nanowires^6^, thin films^7–14^, and systems with a finite thickness^15,16^. On the other hand, theoretical and numerical investigations of the impact of the specific sample shape on the magnetisation reversal processes are still in their infancy^17–20^. The domain structure in these materials is primarily related to the intrinsic disorder that contributes to the enhanced stochasticity of the DW motion^21–25^, but this remains poorly understood.

One of the key sources of the DW stochasticity are the dynamic critical fluctuations, which have no particular scale^26^. These occur close to a critical disorder line that separates two distinct dynamical regimes: on one side a weak pinning regime with large propagating domains, and on the other side a strong disorder regime with pinned domain walls and smaller domains. In this context, the changing sample shape and dimensionality can affect the extension of the domains in one or more directions and thus alter the effects of disorder on the domain wall propagation. Consequently, the critical disorder separating the two dynamical regimes can vary with the sample shape and the effective dimensionality. More precise theoretical investigations using the numerical studies of Ising spin model systems with the random-field magnetic disorder (RFIM) and the concept of finite-size scaling^27^ determine the critical disorder ${R}_{c}^{3D}=2.16$ in the three-dimensional^28–30^, and ${R}_{C}^{2D}=0.54$ in two-dimensional systems^31,32^, augmenting earlier studies with a built-in DW^33–35^. Recently^20^, using the extensive simulations and extending the finite-size scaling for the systems with the base *L* × *L* and finite thickness *l*, the critical disorder line ${R}_{c}^{eff}(l,L)$ has been determined interpolating from the two-dimensional ($l=1$) and three-dimensional ($l=L$) RFIM systems. Apart from the value of the critical disorder, the DW motion at different spatiotemporal scales^36,37^ as well as the interplay of the critical fluctuations and the shape of the lattice constitute the theoretically challenging problems of broad importance. A review of some recent developments in classical and quantum systems can be found in^38,39^. Some of the considersations in this paper are reminiscent of research that considers criticality of spin systems situated on a complex network topology^40–43^.

In this work, we tackle some of these issues through the numerical study of magnetisation reversal processes in RFIM systems of variable thickness on the critical disorder line, moving from a two-dimensional plane to the three-dimensional lattice. At one end of this critical line, in the three-dimensional limit, the hysteresis-loop behaviour was investigated extensively by numerical methods^28–30^. The three-dimensional model is also accessible to field-theory approaches^26,44–47^. The renormalization-group approach in another class of models^48–52^ focuses on the criticality of the driven interface depinning in random 3D and 2D systems. In the course of the reversal process along the hysteresis loop, the occurrence of large domain walls and their motion in the central part of the loop, where the external field is close to the coercive field *H*_*c*_, play a crucial role in the critical dynamics. It has been recognised^47^ that in the metastable states near *H*_*c*_ particular configurations of the disorder can trigger a large system-wide avalanche. In contrast, much less is known about the structure of such states in finite geometry samples or in the two-dimensional limit, which appears to be the lower critical dimensionality of the field-theory model. Recent numerical investigations^18–20,32^ indicate a rich dynamical critical behaviour, prone to the impact of geometry and disorder. Therefore, we adopt an adiabatic driving mode, where the field increments adjust to the current minimal local field (see Model and Methods) and focus on the nature of fluctuations in the central part of the hysteresis loop. Our analysis reveals that the samples of sufficiently small thickness have a new critical behaviour on the hysteresis loop, which is different from the one in the two-dimensional limit; these differences manifest themselves at the level of multi-fractality of the Barkhausen noise signal as well as the avalanches of domain-wall slides. On the other hand, the hysteresis-loop criticality in substantially thick samples gradually changes with the increased thickness, increasingly resembling the three-dimensional system.

## Model and Methods

### Field-driven spin reversal dynamics in RFIM

Random-field disorder, which locally breaks the rotational symmetry of the order parameter, is considered to adequately describe the impact of magnetic defects on criticality in classical^53^ and quantum^54^ spin systems. To model the effects of disorder on the magnetisation reversal along the hysteresis loop, we use a RFIM driven by the time-varying external field *H*_*ext*_ at zero temperature^53,55,56^. The Hamiltonian of interacting Ising spins ${s}_{i}=\pm \,1$ is

1 $$ {\mathcal H} =-\,J\,\sum _{\langle i,j\rangle }\,{s}_{i}{s}_{j}-\sum _{i}\,{h}_{i}{s}_{i}-{H}_{ext}\,\sum _{i}\,{s}_{i},$$

where $i=1,2,\ldots N$ runs over all sites and ${\sum }_{\langle i,j\rangle }$ denotes the summation over all pairs of nearest neighbour spins on the lattice of the specified size *L* × *L* × *l*, and the strength of the ferromagnetic coupling is fixed $J=1$. At each lattice site, the value *h*_*i*_ of the random field is chosen from the Gaussian distribution $\rho (h)={e}^{-{h}^{2}/2{R}^{2}}/\sqrt{2\pi }R$ of zero mean and the variance $\langle {h}_{i}{h}_{j}\rangle ={\delta }_{i,j}{R}^{2}$. The realisation of these random fields is considered as a quenched disorder^53^, consequently, the fields are kept fixed during the system’s evolution. The deterministic (zero-temperature type) dynamics consists of spin-flip ${s}_{i}(t+1)=-\,{s}_{i}(t)$ by aligning the spin *s*_*i*_ with its local field ${h}_{i}^{loc}$, which is given by ${h}_{i}^{loc}=J\,{\sum }_{j}\,{s}_{j}+{H}_{ext}+{h}_{i}$. Apart from the fixed random field *h*_*i*_ at that site, the time varying contributions to ${h}_{i}^{loc}$ are due to the state of all neighbouring spins *s*_*j*_ and the actual value of the external field *H*_*ext*_. The spin system is driven along the ascending branch of the hysteresis loop starting from the uniform state $\{{s}_{i}=-\,1\}$ for all lattice sites, and a large negative *H*_*ext*_. The external field is increased for a small value to start a new avalanche (see below) and the updated values of ${h}_{i}^{loc}$ at all sites are computed and kept until all unstable spins are flipped in the current time step. Then the set of new local fields ${h}_{i}^{loc}$ is determined at sites in the shell at the avalanche front, and the unstable spins flipped; the process is repeated until no more unstable spins can be found. Then the external field is increased again. Note that the number of chain events strongly depends on the state of the system, the strength of disorder *R*, and the actual value of the external field, i.e., the hysteresis loop segment.

The sequence of spin-flip events between the two consecutive updates of the external field comprise an *avalanche*. This larger-scale event can be characterised by the duration *T*—the number of time steps, and size *S*—the number of flipped spins during the avalanche propagation, i.e., $S={\sum }_{t={t}_{s}}^{{t}_{e}}\,{n}_{t}$ and $T={t}_{e}-{t}_{s}$, where *n*_*t*_ is the number of spins flipped during the step *t*, and *t*_*s*_ and *t*_*e*_ indicate the moments when the avalanche begins and ends, respectively. Note that in the zero-temperature dynamics the number *n*_*t*_ gives the exact change of the magnetisation $\delta M(t)\equiv M(t+1)-M(t)=2{n}_{t}/N$ at time *t*, constituting the time signal known as Barkhausen noise. Here, the magnetisation $M(t)={\sum }_{i=1}^{N}\,{s}_{i}/N$ varies with *t* depending on the state of all spins. To minimally affect the avalanche propagation the driving field is incremented *adiabatically*, that is, the external field *H*_*ext*_ is held constant during each avalanche. Moreover, the field that starts a new avalanche is updated by the amount that matches the local field of the minimally stable spin in the entire system, which is identified using a sorted-list search method^55^. The process ends when all spins are reversed completing the hysteresis branch. We sample two sets of systems of the size *L* × *L* × *l* where the thickness $l={2}^{k}$, $k=0,1,2,3,\ldots$, i.e., from $l=1$ corresponding to the two-dimensional x-y plane of the size *L* × *L* until $l=L$ complete three-dimensional sample. The linear size *L* of the considered systems are $L=256$ and $L=512$. The periodic boundary conditions are applied along x-y directions while the open boundaries are kept in the perpendicular direction of changing thickness. For each system, we sample the number of flipped spins {*n*_*t*_} along the entire branch of the hysteresis loop (Barkhausen noise signal), and identify each avalanche that occurred during the full magnetisation reversal. To complete the avalanche statistics, we repeat the process by new samples of the random fields with the same disorder strength *R*. The number of runs per one set of *l* and *L* pair, performed at the corresponding value of effective critical disorder ${R}_{c}^{eff}(l,L)$, ranged between 500 for the large, and 60 000 for the small samples. The sorted-lists algorithm is very efficient. For the largest system simulated in this work, the single run time on the Supermicro server 8047R-7RTF+ is about 5 hours.

### Critical disorder of systems with finite thickness

The critical fluctuations comprise of the avalanches of all sizes including an infinite system-size avalanche. In the finite-size *L* systems with periodic boundary conditions, these are represented by *spanning* avalanches that occur at an effective critical point ${R}_{c}^{eff}(L)$. Then the true critical point ${R}_{c}(L\to \infty )$ is extracted by applying the finite-size scaling collapse^27^. Using these ideas and simulations of the RFIM in very large systems, the critical disorder has been determined as ${R}_{c}^{3D}=2.16\pm 0.06$ in $D=3$, and ${R}_{c}^{2D}=0.54\pm 0.06$ in $D=2$ spatial dimensions^28–30,57^. In the finite-size scaling spirit, a system of finite thickness exhibits critical fluctuations for a reduced disorder compared with the full three-dimensional geometry. Recently, extensive simulations and the finite-size scaling analysis of avalanches for the systems of size *L* × *L* × *l* with varied thickness *l* have been performed in^20^. In this case, the spanning avalanches in the x-y dimensions are relevant in addition to the extra scaling variable *l*/*L*, due to the open boundaries in the *l*-direction. The analysis led to the critical disorder line

2 $${R}_{c}(l)=\frac{{R}_{c}^{3D}}{1-{\rm{\Delta }}/{l}^{1/{\nu }_{3D}}},$$

where ${\rm{\Delta }}=1-{R}_{c}^{3D}/{R}_{c}^{2D}$ and ${\nu }_{3D}$ is the correlation-length exponent of the corresponding three-dimensional system. Relevant for this work is the effective critical disorder of the system of the finite base length *L* and thickness *l* that can be obtained from the analysis in^20^, in particular:

3 $$\frac{{R}_{c}^{eff}(l,L)-{R}_{c}(l)}{{R}_{c}^{eff}(l,L)}=\frac{A(l)}{{L}^{1/{\nu }_{2D}}}$$

where *A*(*l*) was shown to scale with the thickness as $A(l)=\frac{(a-{\rm{\Delta }}){l}^{1/{\nu }_{2D}}}{{l}^{1/{\nu }_{3D}}-{\rm{\Delta }}}$ and $a=0.63\pm 0.18$ is the fit parameter. The respective values are 1/${\nu }_{3D}=0.71$ and 1/${\nu }_{2D}=0.19$, using the exponent controlling the divergence of the correlation length for $l=L$ in $D=3$^28^, and $l=1$ in $D=2$ limit^31^.

### Avalanche distributions and average shapes

Regarding the statistics of avalanches at the critical disorder, we distinguish the loop-integrated distributions (int), including the avalanches that appear over the entire branch of the hysteresis, and the distributions of the avalanches occurring only in the central part of the hysteresis loop (HLC). In the limiting 2D and 3D cases, the distributions of the avalanche size *P*(*S*) and duration *P*(*T*) obey power-law decay $P(x,L)=A{x}^{-{\tau }_{x}}{\mathfrak{P}}(x/{L}^{{D}_{x}})$ with a finite size cut-off and corresponding fractal dimension *D*_*x*_, which are well studied in the literature^29,30,57^. For example, for the disorder $R\ge {R}_{c}^{3D}$, the scaling function ${{\mathfrak{P}}}_{+}$ represents a product of a polynomial and a stretched exponential^28,56^; whereas, ${{\mathfrak{P}}}_{-}$ corresponding to disorders $R < {R}_{c}^{3D}$ is further modified to include the spanning avalanches of different dimensions^29,30,57^. In the samples of finite thickness with the lattice size *L* × *L* × *l*, the appearance of the extra scaling variable *l*/*L* induces substantial changes both in the scaling function and the exponents (see Results). In this case, we observe two distinct slopes for small and large avalanches, respectively, which can be fitted by the following expression

4 $$P(S)=\{[1-\,\tanh (S/B)]\frac{{A}_{1}}{{S}^{{\tau }_{1}}}+\,\tanh (S/B)\frac{{A}_{2}}{{S}^{{\tau }_{2}}}\}{\mathfrak{P}}(S),$$

for the avalanche size *S* and ${\mathfrak{P}}(S)$ the scaling function for a particular size *L* and thickness *l*. The factor in the curly brackets in (4) is a convex combination of two power-laws, *A*_1_/${S}^{{\tau }_{1}}$ and *A*_2_/${S}^{{\tau }_{2}}$, specified by the amplitudes *A*_1_ and *A*_2_, and exponents ${\tau }_{1}$ and ${\tau }_{2}$, respectively. For $S\ll B$, the first power-law prevails, so ${\tau }_{1}$ gives the slope of the log-log plot of the curve *P*(*S*) in that region. Then, for $S\approx B$, the distribution curve bends and proceeds with the second slope ${\tau }_{2}$ in the part of scaling region where $S\gg B$, up to the large-avalanche cutoff, where the universal scaling function ${\mathfrak{P}}(S)$ becomes dominant. At disorders above the effective critical disorder, a stretched exponential form ${\mathfrak{P}}(S)=\exp [\,-\,{(S/C)}^{\sigma }]$ can be used. To capture the contribution of different types of spanning avalanches that typically occur at and below the critical disorder, we use a more elaborate expression^58^ ${\mathfrak{P}}(S)=\exp [{(S/D)}^{k}-{(S/C)}^{\sigma }]$. Note that the distribution $P(S)=\tilde{A}{S}^{-\tau }{\mathfrak{P}}(S)$ with a similar form of scaling function was derived in the renormalization group theory for the elastic interface^48–50^, where the exponent *k* = 1/2 is fixed, while the exponents *σ* and $\tau$ as well as the parameters are determined by $\varepsilon$-expansion ($\varepsilon =1$ for the 3D and $\varepsilon =2$ for the 2D case). A similar expression (4) applies for the duration *T* of avalanches, with the corresponding exponents *α*_1_ and *α*_2_ and a scaling function ${\mathfrak{P}}(T)$. The bending value $B={S}_{x}$ of the size and $B={T}_{x}$ of the duration distribution depend on the actual sample thickness (see Results).

The average size of all avalanches of given duration *T*, ${\langle S\rangle }_{T}$, also exhibits a scale invariance ${\langle S\rangle }_{T}\propto {T}^{\gamma }$ with the exponent $\gamma =(\alpha -1)/(\tau -1)$. With two distinct scaling regions in the distributions of size and duration, here also two exponents *γ*_1_ and *γ*_2_ can be observed for some intermediate sample thicknesses. Similarly, two values of *γ* are extracted from the data for the average avalanche shape for small and large durations using the analytical form^59^ (a more general form was derived using the renormalization group methods in^60,61^)

5 $$\langle {n}_{t}(t|T)\rangle \propto {T}^{\gamma -1}{[\frac{t}{T}(1-\frac{t}{T})]}^{\gamma -1}\times [1-a(\frac{t}{T}-\frac{1}{2})].$$

Here, $\langle {n}_{t}(t|T)\rangle$ refers to the number of spins *n*_*t*_ flipped at the moment *t* since the start of the avalanche whose duration is *T* and averaged over all avalanches of the duration *T*. Therefore, in the present context, the quantity *n*_*t*_ measures the pace of propagation of the avalanche front, analogous to the velocity of the interface; the exponent *γ* is defined above and *a* is the asymmetry parameter.

### Detrended multifractal analysis of Barkhausen noise signal

As demonstrated in^36^, the convenient approach of studying the multifractal features of the magnetisation reversal fluctuations exploits the underlying scale-invariance to determine the generalised Hurst exponent *H*(*q*). The respective time series *δM*(*k*), $k=1,2,\ldots {T}_{{\max }}$ of the length *T*_*max*_ comprises a selected segment of the BHN signal {*n*_*t*_} on the hysteresis loop (see Results). Following the standard procedure described in^36,62–64^, the profile of the time series $Y(i)={\sum }_{k=1}^{i}\,(\delta M(k)-\langle \delta M\rangle )$ is firstly obtained, and thereafter divided into non-overlapping segments of equal length *n*. The process is repeated starting from the end of the time series resulting in total $2{N}_{s}=2\,{\rm{int}}({T}_{{\max }}/n)$ segments; here, int(*x*) is the integer part of a real number *x*. Then, the local trend *y*_*μ*_(*i*) is found at each segment $\mu =1,2\ldots {N}_{s}$, which enables the determination of the standard deviation $F(\mu ,n)$ around the local trend

6 $$F(\mu ,n)={\{\frac{1}{n}\sum _{i=1}^{n}{[Y((\mu -1)n+i)-{y}_{\mu }(i)]}^{2}\}}^{1/2},$$

and similarly, $F(\mu ,n)$ = ${\{\tfrac{1}{n}{\sum }_{i=1}^{n}{[Y(N-(\mu -{N}_{s})n+i)-{y}_{\mu }(i)]}^{2}\}}^{1/2}$ for $\mu ={N}_{s}+1,\ldots 2{N}_{s}$. Finally, the *q*-th order fluctuation function *F*_*q*_(*n*) is computed for segment length *n*, and averaged over all segments

7 $${F}_{q}(n)={\{\frac{1}{2{N}_{s}}\sum _{\mu =1}^{2{N}_{s}}{[{F}^{2}(\mu ,n)]}^{q/2}\}}^{1/q}\sim {n}^{H(q)}.$$

The idea behind this formula is that various segments of the signal need to be enhanced in different ways (values of *q*) to achieve a self-similarity of the whole signal. In particular, *small fluctuation segments* are enhanced by the negative values of *q*, while the segments with *large fluctuations* dominate the fluctuation function for the positive values of *q*. By varying the segment lengths in the range $n\in [2,\,{\rm{int}}({T}_{{\max }}/4)]$, we compute the fluctuation function *F*_*q*_(*n*) for different $q\in [\,-\,10,10]$. Plotting *F*_*q*_(*n*) against *n* allows us to find the regions of scale invariance and the corresponding scaling exponent *H*(*q*), as the slope of straight lines in the double-logarithmic plot. Furthermore, the exponent $\tau (q)$ of the box probability measure, standardly defined in the partition function method, is related to *H*(*q*) via the scaling relation $\tau (q)=qH(q)-1$. Hence, the singularity spectrum ${\rm{\Psi }}(\alpha )$ is obtained from *H*(*q*) via the Legendre transform of $\tau (q)$. In particular, ${\rm{\Psi }}(\alpha )=q\alpha -\tau (q)$, where $\alpha =d\tau /dq=H(q)+qdH/dq$. For a monofractal, we have $H(q)=H=const$ and $\alpha =H$; consequently, ${\rm{\Psi }}(\alpha )$ reduces to a single point.

## Results and Discussion

### Hysteresis loop and signal shape in thin samples at the critical disorder

**Figure 1:**
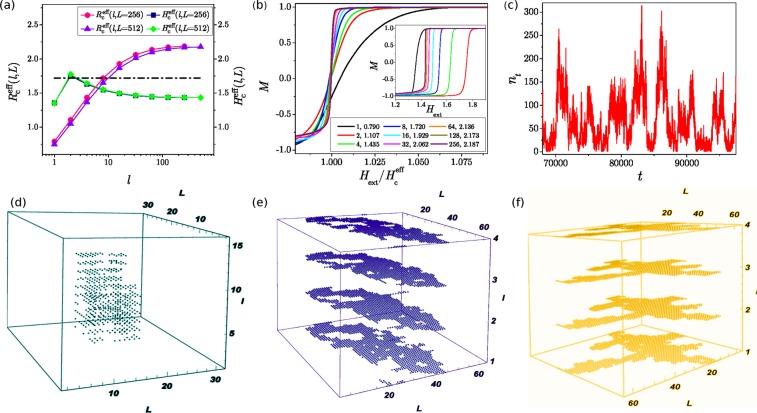
(**a**) Effective critical disorder ${R}_{c}^{eff}(l,L)$ and the coercive field ${H}_{c}^{eff}(l,L)$ versus sample thickness *l*, for the systems with base size $L=256$ and $L=512$. The horizontal line at a fixed disorder is to indicate a typical variation of system parameters accessible to experiments (see Supplementary Information). (**b**) Magnetization *M* against the rescaled magnetic field *H*/${H}_{c}^{eff}$ for various sample thicknesses *l* and base size $L=256$; for each *l* the magnetization curve is obtained at the corresponding effective critical disorder ${R}_{c}^{eff}(l,L)$ shown in the legend. Inset: the same magnetization curves versus the magnetic field *H*. (**c**) An example of the BHN signal *n*_*t*_ against time *t*; the fragment is extracted from the response of a system at the critical disorder ${R}_{c}^{eff}$ for $L=256$ and small thickness $l=4$. (**d**–**f**) Sample avalanches: non-spanning ($R=2.5,l=16,L=32$), 1D-spanning ($R=1.9,l=4,L=64$), and 2D-spanning ($R=1.8,l=4,L=64$), respectively.

The critical disorder line ${R}_{c}^{eff}(l,L)$ for the sample with the base size $L=256$ and varied thickness $l={2}^{k}$, $k=0,1,2,\ldots 8$ is plotted in Fig. 1a together with the effective coercive field ${H}_{c}^{eff}(l,L)$; the corresponding lines for the case of $L=512$ are also shown. The effective critical disorder increases at small thickness and then saturates approaching the values for the 3D samples. As expected in disordered materials^13,53^, the increased disorder induces narrowing of the hysteresis loop, which is compatible with the smaller values of the effective coercive fields ${H}_{c}^{eff}(l)$ for $l\ge 2$, as shown in Fig. 1b.

The small thickness also affects the shape of the signal and the propagation of avalanches, as demonstrated in Fig. 1c–f. As mentioned above, the avalanches of different sizes including the sample-spanning avalanches are expected at critical disorder. In the case of small thickness, the avalanche often hits the system’s open boundary in the z-direction and stops, while the propagation in the x-y directions within the sample is conditioned by the pinning of avalanches by the random-field disorder; some examples of avalanches are shown in Fig. 1e,f. Hence, the sample thickness determines the actual shape of the critical avalanches. These effects are also manifested in the shape of the accompanying BHN signal. For example, for a large and thin sample, see Fig. 1c, small variations of the signal occurring due to pinning at the boundary appear intermittently between the large fluctuations even in the central part of the hysteresis loop. A detailed analysis below reveals how these fluctuations are manifested in the multi-fractal properties of the BHN signal in the thin samples.

**Figure 2:**
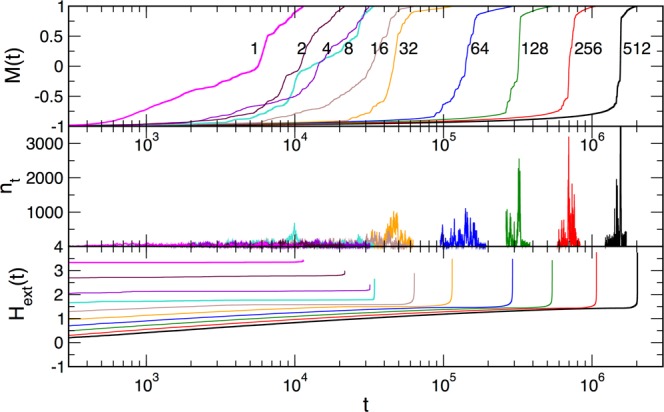
Magnetisation *M*(*t*) plotted against time *t* (top panel), the corresponding BHN signal *n*_*t*_ (middle panel), and the time-varying external field *H*_*ext*_(*t*) (lower panel) for the increasing sample thickness *l*, indicated in the top panel, from 2D sample $l=1$ to 3D sample $l=L=512$. The part of the signal corresponding to the center segment of the hysteresis loop are shown in the middle panel. The logarithmic scale along the time axis is applied. The beginning of the loop is omitted to improve clarity.

Figure 2 shows how the sample thickness affects the magnetization increase with time in the ascending branch of the hysteresis loop. Precisely, the pronounced effects occur in the case of small thickness $l\lesssim {l}_{tr}$, where ${l}_{tr}\approx L/8$ is a transitional thickness, which depends on the base size *L*. In contrast, for the thicker samples with $l > {l}_{tr}$, the effects of the finite thickness are more predictable, as the analysis below will show. The majority of the critical fluctuations permitting the spanning avalanches occur in the central part of the hysteresis loop (HLC); therefore, we mainly focus on these segments of the loop. The corresponding segments of the BHN signal at each sample thickness are indicated in the middle panel of Fig. 2, while the related values of the external fields that cause these fluctuations are given in the lower panel. Note that, due to the adiabatic driving where the field is kept fixed during the avalanche propagation, the effective driving rate in the HLC segments is minimal, thus allowing a spontaneous evolution of the system.

**Figure 3:**
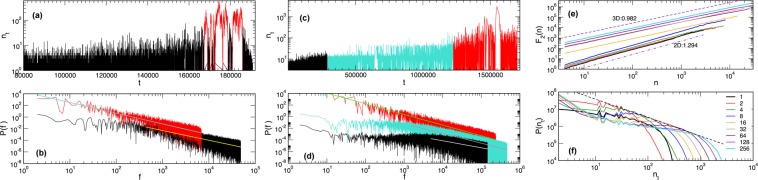
Signal selection according to the avalanche sizes for $L=256$, $l=32$ (**a**,**b**), and according to three hysteresis-loop segments for $l=L=512$ (**c**,**d**). The lower panel in each case shows the corresponding power spectrum of the selected signals against frequency *f* with the slopes $\varphi =1.84\pm 0.02$ and $1.67\pm 0.02$, panel (b), and $\varphi =1.835\pm 0.014$, $1.673\pm 0.008$, and $1.208\pm 0.009$, panel (d). Second-order fluctuation function *F*_2_(*n*) vs segment length *n* for $L=512$ and varied thickness $l=512$ top line to $l=1$ bottom line (**e**); the two dashed lines have the slopes equal to the Hurst exponent *H*(2) in 2D and 3D case. Distributions of the height *n*_*t*_ of the BHN signal for $L=256$ and varied thickness *l* indicated in the legend, and a tangent—dashed line, (**f**).

As mentioned above, the size and thickness of the sample affects the critical disorder ${R}_{c}^{eff}(l,L)$ and, consequently, the shape of the BHN signal. Some features of the BHN signal obtained at the critical disorder in samples of different thickness are illustrated in Fig. 3a–f. The signals exhibit long-range temporal correlations with the power spectrum $P(f)\sim {f}^{-\varphi }$ over an extended range of frequencies *f*. The previous studies of the multifractal features of the BHN in 2D and bulk samples in a strong disorder regime^19,36^, suggest that the signal shape differs in different segments of the hysteresis loop. Here, we demonstrate how the size and temporal correlations of the signal change along the hysteresis loop in the 3D sample at the critical disorder, see Fig. 3c,d. Moreover, in the present context, it is interesting to point out another segmentation of the signal, which comprises the separation of small and large avalanches occurring in a thin sample. Two panels in Fig. 3a,b show the respective separation and the corresponding power spectra for a sample of the transitional thickness *l*_*tr*_(*L*) for the given base size *L* (see below for its precise definition). Furthermore, the persistent fluctuations are observed that are compatible with the Hurst exponent $H(2)\lesssim 1$ in samples of a larger thickness, whereas $H(2) > 1$ for the thin samples having $l < {l}_{tr}(L)$, as shown in Fig. 3e. For the analysis in this paper, it is also important to note that the distribution of the signal heights *n*_*t*_ (data points) shows a broad peak that moves to the right with the increased sample thickness, as shown in Fig. 3f. The tangent line has a power-law slope, while the small signal heights have entirely different distribution, which also varies with the thickness.

### Critical avalanches in samples of different thickness

**Figure 4:**
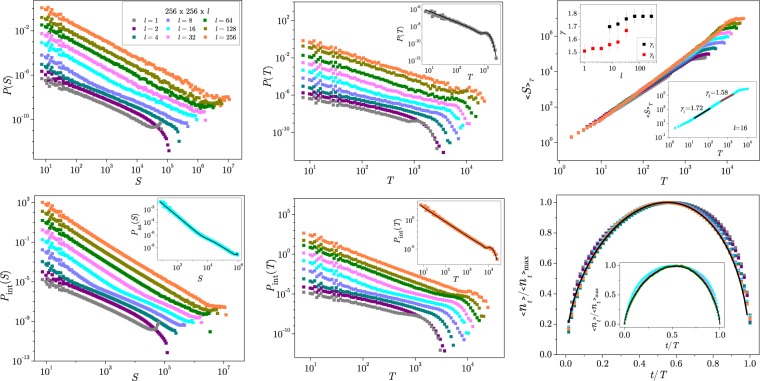
Avalanche distributions for different thicknesses *l* given in the legend (applies to all four panels). Left column: size distributions in the HLC (top), and loop-integrated size distributions (bottom panel, with the best fit of type (4) for $l=16$ in the inset). Middle column: corresponding duration distributions (top panel, with the best fit of type (4) for $l=1$ in the inset), and integrated duration distributions (bottom panel, with the best fit of type (4) for $l=256$ in the inset). Right column: average size of avalanches ${\langle S\rangle }_{T}$ having a particular duration *T*; Insets: determination of the exponents *γ*_1_ and *γ*_2_ (lower-right), and their variation with *l* (top-left inset). Bottom right panel shows the normalised average avalanche shapes $\langle {n}_{t}\rangle$ vs *t*/*T* for various *l* and the fixed duration $T=64$ (main panel) and $T=2048$ (inset). Fits according to (5) with *a* = −0.214, *γ* = 1.51, main panel, and *a* = −0.176, *γ* = 1.628, inset.

In Fig. 4 we show the distributions of size *P*(*S*) and duration *P*(*T*) of the avalanches obtained for various sample thicknesses. These distributions contain avalanches collected in the central part of the hysteresis loop in a small window of the external field, and are most relevant for the critical dynamics. In addition, we also show the results for the avalanches collected along the entire hysteresis branch, denoted by *P*_*int*_(*S*) and *P*_*int*_(*T*), for size and duration, respectively, that are typically determined in the analysis of the experimental BHN signals.

In contrast to the avalanches in strictly two-dimensional^31,32^ and three-dimensional RFIM^29,30,56^, the avalanche distributions in the samples of finite thickness exhibit two distinct scaling regions, for small and large avalanches, respectively, as shown in Fig. 4. More specifically, the first larger slope (identified by the exponent ${\tau }_{1}$ and similarly *α*_1_, see Model and Methods) describes the scale-invariant behaviour of small avalanches. Whereas the second region with a smaller exponent ${\tau }_{2}$ (and *α*_2_) relates to the avalanches larger than the bending size *S*_*x*_ (or duration *T*_*x*_). The bending point *S*_*x*_ (and corresponding *T*_*x*_) depends on the sample thickness and the base size, and gradually moves towards larger size with an increased sample thickness. Thus, we find that the larger slope appears and can be measured for the lattices of quite small thickness; it gradually wins, and when $l\to L$ approaches the exponent ${\tau }_{1}\to {\tau }^{3D}$ and ${\alpha }_{1}\to {\alpha }^{3D}$ (note open boundary conditions). The two-slope distributions are typically found for sufficiently thin samples, i.e., $8\le l\le 32$ for $L=256$, and $16\le l\le 64$ for $L=512$ (see also Fig. 3e). It should be stressed that these features apply to both the loop-integrated avalanches as well as the avalanches in the central part of the hysteresis, as also demonstrated in Fig. 4. Therefore, although the corresponding exponents are somewhat smaller in the central hysteresis segment, the occurrence of two scaling regions in the distributions of the critical Barkhausen avalanches is a unique property of the thin samples with $l\lesssim {l}_{tr}$. According to these results (see also the discussion on multifractality below), the transient thickness can be estimated as ${l}_{tr}\approx L$/8 above which the system effectively behaves as a thick sample.

**Figure 5:**
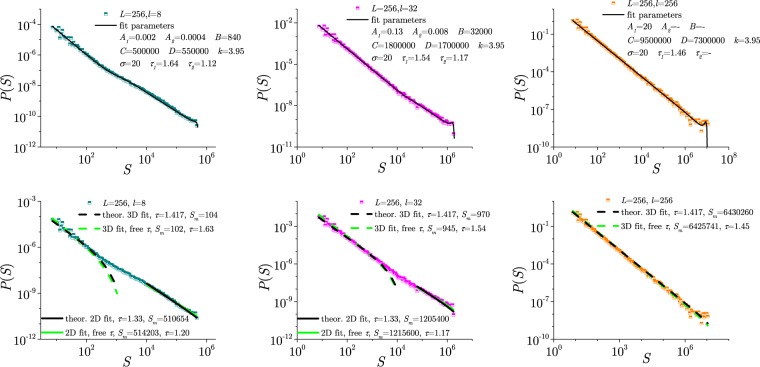
In the central part of the hysteresis loop, the distribution of avalanche size *P*(*S*) for different thickness *l* = 8, 32, and 256 is fitted using the expression (4), top row, and the theoretical expression predicted for interface dynamics^48–50^, where 2D and 3D parts of the distribution are fitted separately, bottom row.

Moreover, our findings indicate that the bending size scales as ${S}_{x}\propto {l}^{{D}_{f}}$, where ${D}_{f}=2.78$ is the fractal dimension of nonspanning avalanches in three dimensions^29,30,56^. Similarly, for the duration distributions, the bending duration ${T}_{x}\propto {l}^{{z}_{d}}$, where ${z}_{d}=1.7$ is the dynamical critical exponent of the 3D model, describing the scaling of the avalanche’s duration with the linear size. For samples of different thickness, the two sets of exponents, i.e., ${\tau }_{1}$ and ${\tau }_{2}$ that describe two distinct power-law regions of the distribution of avalanche size, and the corresponding exponents *α*_1_ and *α*_2_ of the avalanche duration were determined by fitting the entire distribution using the expression (4) proposed in Model and Methods (see Supplementary Information, Fig. SI-1). The estimated values of the exponents ${\tau }_{1}$ and *α*_1_ and ${\tau }_{2}$ and *α*_2_ are summarised in Table SI-1 in the Supplementary Information both for the distributions in the central hysteresis part and the loop-integrated distributions. For the distributions in the HLC, where extended domain walls can occur, it is tempting to fit the simulated data with the expression derived in the RG theory of the interface motion. A more detailed description is given in Supplementary Information. Some representative examples of such fits are given in the bottom row in Fig. 5, while the corresponding fits with the expression (4) are shown in the top row. The theoretical distribution for $\varepsilon =1$ gives a satisfactory fit in the full 3D sample, and similarly, the expression for $\varepsilon =2$ in the 2D limit. However, for the samples of finite thickness, the two expressions must be used separately to fit the first (3D) part and the second (2D) part of the distribution. Moreover, these analytical expressions do not take into account the variation of the scaling exponents with thickness and the precise form of the scaling function.

The appearance of two scaling regions in the avalanche distributions manifests in the plots of the average size ${\langle S\rangle }_{T}$ of avalanches having duration *T*, shown in the right column of Fig. 4 (top panel), and the average avalanche shapes, (bottom panel). The corresponding scaling exponent *γ*, defined via ${\langle S\rangle }_{T}\sim {T}^{\gamma }$ (see Model and Methods), also appears to have distinct values *γ*_1_ for small, and *γ*_2_ for large avalanches at the intermediate thickness $l\le {l}_{tr}$, see the top inset. Within the error bars, the estimated values fall in the range ${\gamma }_{1}=1.73\pm 0.05$ for large *l*, which agrees with the value found in the case of the equal sized 3D cubic lattices (${\gamma }_{3D}=1.73$), while the values for ${\gamma }_{2}=1.56\pm 0.06$ are lower, and close to the case in 2D square lattices (${\gamma }_{2D}=1.55$). The average avalanche shapes collected from all sample thicknesses appear to be asymmetric, see the lower-right panel of Fig. 4. The longer avalanches appear to be more symmetrical and the value for *γ* estimated using the expression (5) is bigger compared with the shape parameters of the short avalanches. In Supplementary Information in Table SI-2, we show the values of the asymmetry parameter *a* along with the exponent *γ* that are computed^58^ for different sample thicknesses and a wide range of the avalanche duration $T\in [64,2048]$. Remarkably, the values of the asymmetry parameter *a* are negative for all thicknesses, following the predictions of RG theory^60^.

### Multiscale multifractality of the critical BHN signal

**Figure 6:**
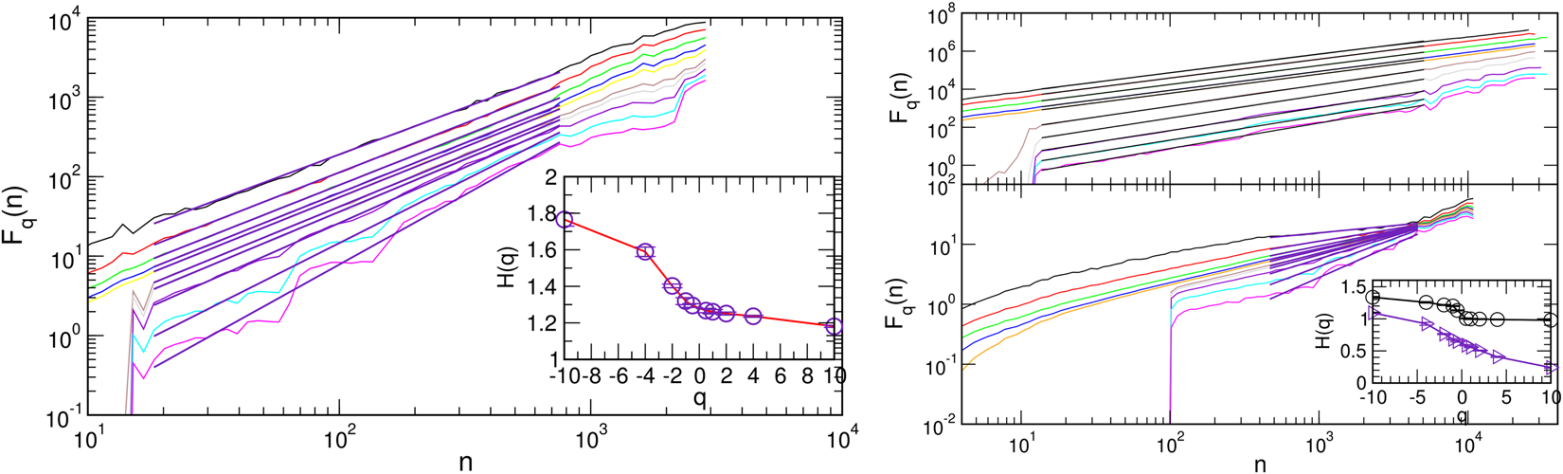
Fluctuation function ${F}_{q}(n)$ for different values of $q\in [\,-\,10,10]$ for the whole signal for the 2D sample of size $L=512$ (left panel), and 3D sample in the HLC (right, upper panel) and the initial segment of the loop (right, lower panel). Insets: corresponding generalised Hurst exponents *H*(*q*) against the amplification parameter *q*, see text.

The properties of BHN signal at different sample thicknesses in Fig. 3e suggest that the magnetisation fluctuations are persistent with the (standard deviation) Hurst exponent varying between $H(2)\lesssim 1$ in 3D samples to $H(2)\approx 1.29$ in the 2D case. To understand the impact of the sample thickness on the multifractal features of BHN signals, we first analyse the two limiting cases. The fluctuation function *F*_*q*_(*n*), defined by (7) in Model and Methods, is computed for the samples of size $L=512$ with $l=1$ (2D sample) and $l=L$ (3D sample), and shown in Fig. 6.

In the 2D limit, the scale invariance of the fluctuation function *F*_*q*_(*n*), c.f. left panel in Fig. 6, shows that the whole signal exhibits multifractal properties for a broad range of time scales *n* with the generalised Hurst exponents $H(q)\in [1.2,1.8]$, shown in the inset. For the 3D case, however, the signal in different segments of the hysteresis loop exhibits different features, see also Fig. 3c,d for the signal segments and their power spectra. Specifically, in agreement with previous studies^36^, the signal in the central segment of the loop has $H(q) > 1$, while the fluctuations at the very beginning of the loop are governed by the actual random field distribution, resulting in a fractional Gaussian type of noise (fGN); consequently, its multifractal spectrum remains in the range $H(q) < 1$. For both cases the exponents *H*(*q*) are shown in the inset of the right panel of Fig. 6. As stated above, here we focus on the impact of thickness on the critical fluctuations, which are prominent in the central part of the hysteresis loop. A systematic analysis of the entire hysteresis loop for a particular sample shape is left for another study.

**Figure 7:**
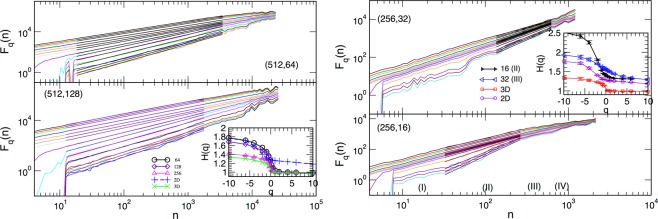
Fluctuation function ${F}_{q}(n)$ for $q\in [\,-\,10,10]$ computed in the central part of the hysteresis loop for samples of different base size *L* and thickness *l*; each pair (*L*, *l*) is indicated in the corresponding panel. Straight lines indicate the fitted scaling regions, and the corresponding generalised Hurst exponents *H*(*q*) are plotted against *q* in the insets. See text for more details.

In the finite samples, the spectrum *H*(*q*) changes, depending on the ratio *l*/*L* of the thickness *l* relative to the base size *L* of the system, see Fig. 7. Our numerical analysis suggests that the most dramatic changes occur in small fluctuations region ($q < 0$) and when the samples are sufficiently thin such that $l/L\lesssim 1/16$. More specifically, for relatively thick samples with $l/L\ge 1/8$, left panels in Fig. 7 show that the multifractal features are apparent in a broad range of time scales *n*. For $q > 0$, the exponents *H*(*q*) remain in the area of the standard Hurst exponent *H*(2) of a 3D sample, whereas significant deviations occur in $q < 0$ region, governing small fluctuations. This part of spectrum gradually approaches the one observed in the bulk 3D samples when $l\to L$, as shown in the inset.

**Figure 8:**
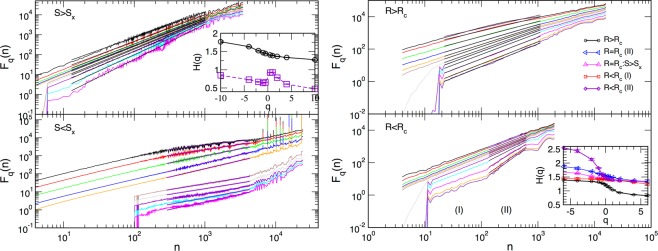
For the sample of base size $L=256$ and thickness $l=32$, the fluctuation function *F*_*q*_(*n*) of the signal segments selected according to the avalanches above (below) the bending size *S*_*x*_, left panels, and signals in the HLC for the disorder above (below) the effective critical disorder, right panels, denoted as *R*_*c*_. Corresponding generalised Hurst exponents *H*(*q*) are shown against *q* in the respective insets.

**Figure 9:**
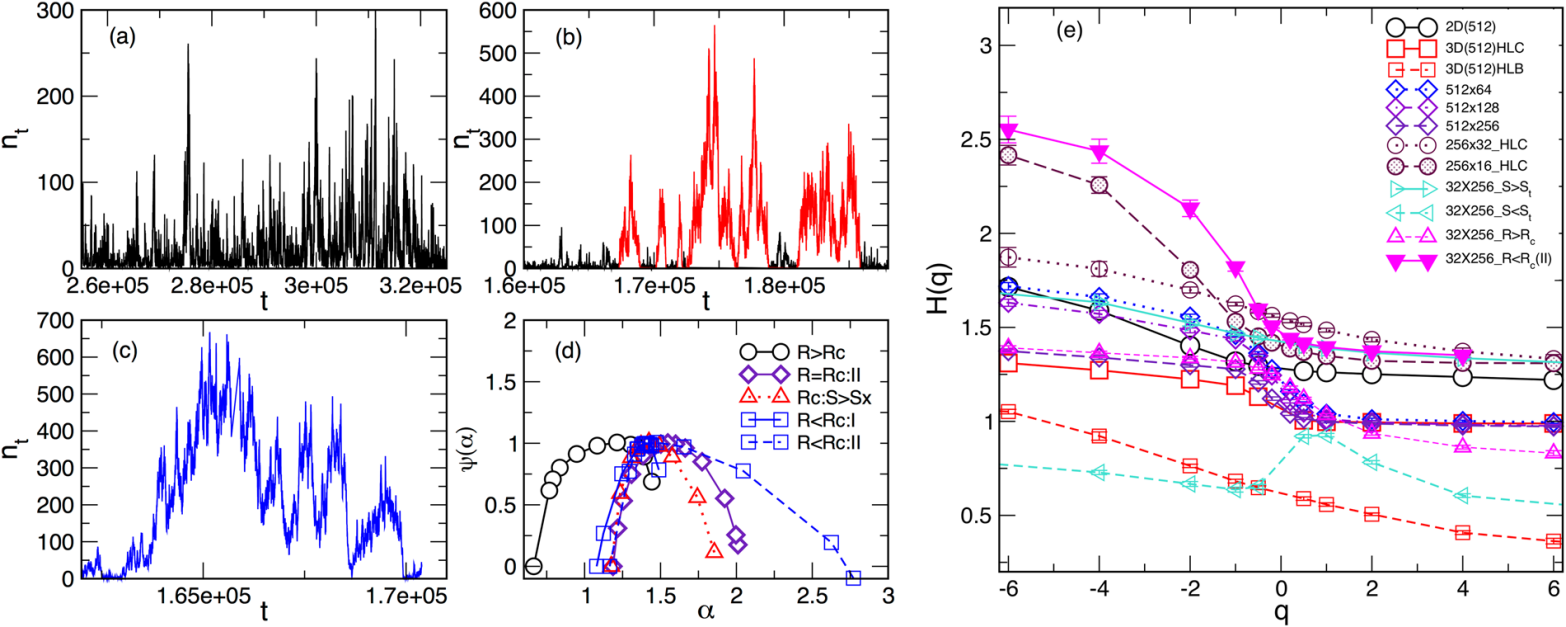
(**a**–**c**) Noise signal in the central part of HL for $R > {R}_{c}$, $R={R}_{c}$, and $R < {R}_{c}$ at the transitional sample thickness ${l}_{tr}=32$ for $L=256$ and the corresponding effective critical disorder, for simplicity denoted by *R*_*c*_. (**d**) The singularity spectra corresponding to these signals and indicated interval range, together with the spectrum referring to large avalanches selection, $S > {S}_{x}$. (**e**) Generalised Hurst exponents *H*(*q*) against *q* for all signals studied in Figs 6, 7 and 8.

On the other hand, the thin samples with $l/L\lesssim 1/16$ exhibit a time-scale dependent behaviour of the fluctuation function *F*_*q*_(*n*), c.f. right panels in Fig. 7. Here, we find that several scaling regions occur, indicated by (I)–(IV) in the lower right panel, where different spectrum *H*(*q*) can be determined. While the multifractality in some of these regions is apparent (see, for example, the region (II) in Fig. 7), some of the other areas appear to have a narrow spectrum which is virtually monofractal, see, for example region (I). In all cases, the values of the generalised Hurst exponents, shown in the inset, are in the range above the corresponding values in the 2D limit. Again, the most significant deviations occur in the negative part of the spectrum $q < 0$. See further discussion and Figs 8 and 9 in the next section.

## Discussion

Our study of the magnetisation reversal processes *along the effective critical disorder line* ${R}_{c}^{eff}(l,L)$ revealed that the pinning of DW at the open boundaries in the direction of small thickness can hinder the propagation of avalanches and the shape of the BHN signal at all scales. For example, in the sufficiently thin samples, many small avalanches can occur whose scaling exponents are close to the 3D RFIM class, at the same time, large avalanches with the size above the bending size *S*_*x*_(*l*), manage to propagate in the two transverse directions, resembling quasi-2D avalanches. The relative contribution of these two types of avalanches to the whole process gradually changes as the sample thickness *l* increases. Consequently, the fluctuations of the magnetisation in the central part of the hysteresis loop changes with the increased thickness. More specifically, for the thick samples, $l\gtrsim {l}_{tr}\sim L/8$, the fractality of the significant fluctuations ($q > 0$) in the HLC virtually coincides with the spectrum of 3D samples, whereas the part of the spectrum with the dominant small fluctuations ($q < 0$) varies, interpolating from the 2D to the 3D case with increasing thickness. On the other hand, in the non-central parts of the hysteresis loop (excluding the very beginning, where all signals are fGN type) and along the whole hysteresis branch of thin samples ($l < {l}_{tr}$), the large and small avalanches intermittently occur, leading to a more complex behaviour of the fluctuation function. Consequently, the generalised Hurst exponent dependence on the time scale (interval length) can be observed. Interestingly, the intervals where multifractal features are apparent roughly coincide with the parts of the signal that are dominated by the large quasi-2D avalanches (see Fig. 8 and the discussion below); in both cases, the generalised Hurst exponents $H(q) > 1$, and is close to the 2D sample spectrum.

To further support these findings, we selected the segments of the signal that correspond to large (small) avalanches, where the bending point *S*_*x*_(*l*) is taken from the corresponding distribution of avalanche sizes, c.f. Fig. 4. An example of the signal selection for $L=256$ and $l=32$ in shown in Fig. 9b. The fluctuation function corresponding to the separate analysis of these parts of the signal is given in left panels of Fig. 8. The large avalanches, which mostly occur in the center of the hysteresis loop, contribute to the leading multifractal spectrum with $H(q) > 1$, see inset in Fig. 8 for $S > {S}_{x}$. Small avalanches, however, exhibit more complex behaviour resulting in several regions with different scaling of the fluctuation function. For instance, ${F}_{q}(n)$ in the intermediate-scale region, marked in Fig. 8 for $S < {S}_{x}$, shows different slopes than the two adjacent regions. Moreover, the smaller slopes of the curves for $q < 0$ compared to $q > 0$ results in a non-smooth spectrum *H*(*q*), also shown in the inset above. Thus, the number of small avalanches that occur due to pinning of the domain walls at the open boundary in samples of small thickness can lead to the observed multi-scale multifractality of these signals.

Next, we investigate whether these features of the BHN signal are exclusively related to the critical avalanches. We perform simulations of the magnetisation reversal in several disorders $R\ne {R}_{c}^{eff}(l,L)$ sightly above the effective critical disorder, $R > {R}_{c}^{eff}(l,L)$, and slightly below it. The corresponding fluctuation functions ${F}_{q}(n)$ are given in the right panels of Fig. 8 for the sample of transitional thickness ${l}_{tr}/L=1/8$ and $L=256$. The related signal shapes and the singularity spectra ${\rm{\Psi }}(\alpha )$ are given in Fig. 9. While the relative size of the time scale changes compared to the critical fluctuations, the scale-dependent multifractality of the signal clearly persists for stronger disorder $R\gtrsim {R}_{c}^{eff}({l}_{tr},L)$. Here, although all avalanches are smaller than the ones at the critical disorder, the co-occurrence of small and large avalanches can be distinguished both in the signal, see Fig. 9a, and in the avalanche distributions (see Supplementary Information, Fig. SI-4). Below ${R}_{c}^{eff}(l,L)$, however, the extended range of the time intervals with virtually monofractal behaviour appears, region (I), shifting the range of the apparent MFR towards larger time scale, region (II), c.f. Fig. 8 lower right panel. At such disorders, a huge avalanche of a prolonged duration appears, as shown in Fig. 9c, whose shape differs from the typical sharply-cut avalanches seen in the case of periodic boundaries that allow depinning of a DW. It is interesting to note that the left part of the singularity spectrum ${\rm{\Psi }}(\alpha )$, which is associated with the large magnetisation fluctuations in this signal, coincides with the corresponding spectrum of the critical fluctuations and its part containing the selected large avalanches. The right parts of these spectra, representing small fluctuations (negative *q*) are different in each of these cases, see Fig. 9d. Compared to these, the spectrum ${\rm{\Psi }}(\alpha )$ for the case $R > {R}_{c}^{eff}(l,L)$ is shifted towards the smaller values $\alpha  < 1$, influenced by the fGN signal in the strong-disorder regime. For comparisons, the spectra *H*(*q*) of all studied signals are summarised in Fig. 9e.

As mentioned earlier, there is considerable interest in the experimental investigations of Barkhausen noise in thin films and samples of different thickness, e.g.^7,9–16^. The behaviour of Barkhausen avalanches observed in these systems depends on the sample composition, driving mode, and the segment of the hysteresis loop where the analysed signal originates, as well as the sample thickness. The alloys N*i*_*x*_*Fe*_1−*x*_ are often studied with a fixed $x\approx 0.8$^9,15,16^ or variable $x\in [0,0.5]$ composition^11,12^ as a good system where the properties of Barkhausen avalanches can be changed by varying the thickness and composition. It should be noted that in contrast to the adiabatic driving used in the numerical investigations, the experimental studies, for example in^15,16^, are performed with a *finite sweep rate* of the applied magnetic field. Moreover, samples of various thicknesses are prepared by the same method and, presumably, have some *constant disorder*, which is difficult to quantify, but will presumably depend on the composition and type of the alloy. In contrast, methods for modifying the disorder^8^ are developed in^11,12^ for films of a constant small thickness, and the domain walls are directly monitored in response to a fixed field. On the other hand, theoretical studies use simplifying models that can describe certain universal features of the underlying critical phenomena. For example, the renormalization-group studies in^5,61^ attempt to uncover the role of the depinning transition in the statistics and propagation of Barkhausen avalanches. In Supplementary Information, in Fig. SI-2 and Table SI-2, we have shown how the RG theory^48–50,60^ for the interface dynamics can elucidate the nature of the asymmetry of avalanche shapes as well as to describe the scaling form of the avalanche size in the limiting 3D and 2D cases. Without an additional scaling field, however, the existing theoretical results cannot be directly extended to the avalanche distribution in samples of finite thickness; here, the *two-dimensional and three-dimensional segments* of avalanches coexist, resulting in variations of the exponents with thickness and a different scaling function.

If we suppose that the RFIM captures the scaling features of the Barkhausen avalanches in these disordered ferromagnets on the hysteresis loop, it is tempting to consider the available experimental results in view of our numerical investigation. In the theoretical phase diagram showing the critical line of the effective RFIM disorder at a finite thickness *l* relative to the bulk sample, ${R}_{c}^{eff}(l,L)/{R}_{c}^{3D}$ vs *l*/*l*_*ref*_, see Fig. SI-5 in Supplementary Information (SI), the above mentioned experimental situations comprise of a horizontal line at a fixed disorder, or a vertical line at a fixed thickness. Each of these lines intersects with the critical disorder line at a particular point, as illustrated in Fig. SI-5. Theoretically, the *change of the scaling behaviour occurs at the point where the critical line is crossed*. Thus, for the thin samples left (above) the critical line, the actual disorder appears to be stronger than the critical, while in the thicker samples on the right (below) the critical line, the disorder is weak, permitting large system-size avalanches due to DW depinning that may occur in the inner part of the hysteresis loop. A more detailed *comparison of the avalanche exponents* measured in the crystalline samples Ni_0.79_Fe_0.21_ of different thickness in^16^ suggests that the potential constant disorder line intersects the critical line at a point corresponding to a thickness 100 nm, leaving 2× and 5× thinner samples on the left of the critical line. Since none of these samples are infinitely thin, the corresponding line should be high enough in the phase diagram, for example, such that the theoretical critical thickness can be close to the theoretical ${l}_{ref}\approx L/16$, as illustrated in Fig. SI-5 in SI. Note that these quantitative comparisons serve only as the RFIM description of the actual intrinsic disorder in that sample, in view of the observed change of the scaling exponents. Then observing that the reference thickness corresponds to 100 nm in these samples, we can place all other experimental data relative to this point, see Fig. SI-5 in SI. Hence, for the two thinner samples, the measured exponents should be dominated by the second slopes $({\tau }_{2},{\alpha }_{2})$; note that the measured values are in agreement with the theoretical ones shown in the Table SI-1 for the loop-integrated distributions. Then for the thicker samples, the exponents of the first slope $({\tau }_{1},{\alpha }_{1})$ and in the central hysteresis loop seem to dominate the observed experimental distributions. Note that in this region, the distance between the critical line and the considered fixed disorder line is rather small; the corresponding theoretical exponents are also highlighted in the Table SI-1, for better comparisons. The simulated avalanche distributions along the fixed disorder line are also shown in Fig. SI-3a,b in SI; in this case, the second slope, which is apparent in the critical avalanches, is practically lost in the sub-critical disorder because of a large number of system-size avalanches (resulting in the peak at the end of the distribution). In the amorphous samples, however, the apparent disorder line seems to be even higher, see Fig. SI-5 in SI. The exponents thus coincide with the ones $({\tau }_{2},{\alpha }_{2})$ up to 2 × *l*_*ref*_, see Table SI-1. Note also that the exponents in^11^ measured for the very thin films of varied composition $x < 0.5$, including $x=0$, are close to the second slopes $({\tau }_{2},{\alpha }_{2})$ estimated in the hysteresis loop centre, which are listed in top-right part of the Table SI-1. More experimental results shown in Fig. SI-5 in SI also confirm this systematic pattern of the avalanche statistics. Moreover, our results suggest that in such thin samples at and around the critical disorder the multi-fractal features of the BHN signal change with the sample thickness and that they can depend on the time interval in which the scaling region is considered. At the critical disorder line, some intervals have virtually mono-fractal behaviour, at the same time, the surrounding intervals can show apparent multi-fractality.

It should be stressed that the applications of the RFIM with the ferromagnetic interactions are limited to systems with strong anisotropy, resulting in collinear spins and narrow domain walls. Note, however, that non-collinear spin configurations appear due to topological frustrations in the case of anti-ferromagnetic interactions on a complex geometry^41^. In ferromagnets on compact lattices, non-collinear spins are naturally described by vector spin models; they also allow the occurrence of thick domain walls with an internal structure, which can affect the domain-wall propagation^65^. Another issue concerns the role of thermal fluctuations in Barkhausen avalanches. As the critical temperature of the studied ferromagnetic alloys is much higher than the room temperature, it is widely accepted that the deterministic (zero-temperature) dynamics suffices to describe the spin reversal process in bulk materials. However, the potential temperature impact on disorder-induced critical fluctuations in thin samples remains an open question for a future study.

## Conclusions

We have demonstrated that a new type of collective dynamics can arise on the hysteresis loop due to the interplay of the sample geometry and critical fluctuations, studied along the critical-disorder line for different thicknesses, interpolating between the strictly two-dimensional and the three-dimensional systems. The geometry of the sample has a profound impact on the magnetic response of sufficiently thin systems, and it is manifested in a time-scale dependent multi-fractality of Barkhausen noise and double power-law distributions of the magnetisation-reversal avalanches, both of which differ from those known in the limiting cases of two-dimensional and three-dimensional geometry. The main cause of these new critical properties can be associated with the pinning of the domain walls at the open boundaries of thin samples, which thus constrain the avalanche shape and its propagation by effectively changing the role of intrinsic disorder, and causes an intermittent appearance of large and small avalanches even in the central segment of the hysteresis loop. These effects are most apparent in the shape of *critical avalanches*, but they can also be observed in the range of disorders close to the critical line. These findings are in agreement with some recent experimental results, in a restricted range of the parameters where the comparison is permitted by given experimentally accessible conditions. In addition to a wide range of samples with different sizes and thicknesses, the presented numerical results include the exact two-dimensional samples and the whole range of time scales, which are beyond the reach of the laboratory experiments. In this regard, our results can serve as a guide for further experimental investigations; they also reveal new features of the domain-wall stochasticity in thin ferromagnetic films, which are important for developing new technological applications.

## Supplementary information

Supplementary Information for The critical Barkhausen avalanches in thin random-field ferromagnets with an open boundary
