# Supplementary material for: The critical Barkhausen avalanches in thin random-field ferromagnets with an open boundary
Source: Sci Rep. 2019 Apr 19;9:6340. doi: 10.1038/s41598-019-42802-w (PMC6474887; doi:10.1038/s41598-019-42802-w)
Supplement: Supplementary file 1 — Supplementary Information for The critical Barkhausen avalanches in thin random-field ferromagnets with an open boundary [file 41598_2019_42802_MOESM1_ESM.pdf]

Supplementary Information for
The critical Barkhausen avalanches in thin random-field ferromagnets with an open boundary

Bosiljka Tadić[1,2], Svetislav Mijatović[3], Sanja Janićević[3], Djordje Spasojević[3], Geoff J. Rodgers[4]
[1]Department for Theoretical Physics, Jožef Stefan Institute, P.O. Box 3000, SI-1001 Ljubljana, Slovenia
[2]Complexity Science Hub, Josefstaedterstrasse 39, Vienna, Austria
[3]Faculty of Physics, University of Belgrade, Belgrade, Serbia
[4]Brunel University London, Uxbridge Middlesex UB8 3PH UK

March 29, 2019

Contents

1 Critical exponents for the entire range of thicknesses 1
 1.1 Table SI-I of all exponents 1
 1.2 Figure SI-1: Examples of fits with two slopes and cutoff 1
2 Comparisons with theoretical predictions 2
 2.1 Fits for the distribution of avalanche sizes 3
 2.2 Shape of avalanches 3
3 Distributions away from the critical line 4
 3.1 Fixed disorder—varied thickness 4
 3.2 Fixed thickness—varied disorder 5
4 Comparisons with experimental results 6

1 Critical exponents for the entire range of thicknesses

To determine the critical exponents in Table SI-I, τ_1, α_1 and τ_2, α_2, representing two slopes of the avalanche size distribution $P(S,l)$ and the duration distribution $P(T,l)$ at different thickness l, we fit the whole distribution using the non-linear weighted least squares method. This gives the best unbiased estimator of the fitting parameters' values provided that the weight of each point is taken as to reciprocal of the variance of that point. We the function (Eq. (4) in Model and Methods):

$$P(S) = \Big\{\big[1-\tanh(S/B)\big]\frac{A_1}{S^{\tau_1}} + \tanh(S/B)\frac{A_2}{S^{\tau_2}}\Big\}\mathfrak{P}(S)\,, \tag{1}$$

where B is the bending size and [5]

$$\mathfrak{P}(S) = \exp[(S/D)^k - (S/C)^\sigma]\,. \tag{2}$$

The simulated data for the avalanche size distributions $P(S,l)$ and $P(T,l)$ both in the hysteresis loop centre and the loop-integrated distributions at different thickness l, see Fig.4 in the paper, are all fitted in this way; a good initial guess for each fitting parameter is very important here, and a lot of attention was dedicated to finding it. The representative fits are illustrated in Fig. SI-1.

1.1 Table SI-I of all exponents

The resulting sets of scaling exponents for the entire range of thicknesses are summarised in Table SI-I. The values of the critical exponents τ_1 and τ_2 for the size distribution and α_1 and α_2 for the duration distribution are shown, estimated from the double power-law and cut-off fits of both loop-integrated and loop-centred avalanche distributions. The corresponding data are shown in the left and the middle column of Figure 4 of the main paper. Empty fields in the Table indicate that the distributions at the corresponding thicknesses follow power-law described not by double, but by a single exponent. The ranges of values shown in different fonts are comparable with the available experimental results, see Fig. SI-5 and the caption for further details.

	loop-integrated: Int				loop-center: HLC			
l	τ_1	α_1	τ_2	α_2	τ_1	α_1	τ_2	α_2
1	-	-	1.41 ± 0.07	1.54 ± 0.04	-	-	1.09 ± 0.04	1.28 ± 0.01
2	-	-	1.36 ± 0.07	1.51 ± 0.02	-	-	1.14 ± 0.03	1.22 ± 0.02
4	-	-	$\mathbf{1.31 \pm 0.10}$	$\mathbf{1.50 \pm 0.03}$	-	-	**1.11**$\pm$0.06	**1.21**$\pm$0.06
8	2.26 ± 0.08	2.98 ± 0.10	$\mathbf{1.30 \pm 0.08}$	$\mathbf{1.47 \pm 0.02}$	1.64 ± 0.06	1.94 ± 0.17	**1.12**$\pm$0.05	**1.24**$\pm$0.02
16	2.11 ± 0.07	2.80 ± 0.15	$\mathbf{1.30 \pm 0.03}$	$\mathbf{1.51 \pm 0.02}$	1.56 ± 0.04	1.90 ± 0.12	**1.12**$\pm$0.03	**1.37**$\pm$0.13
32	2.00 ± 0.06	2.68 ± 0.09	$\mathbf{1.27 \pm 0.02}$	$\mathbf{1.59 \pm 0.02}$	1.54 ± 0.04	1.88 ± 0.07	**1.17**$\pm$0.03	**1.45**$\pm$0.22
64	1.94 ± 0.08	2.60 ± 0.07	-	-	*1.48 ± 0.03*	*1.82 ± 0.07*	-	-
128	1.89 ± 0.05	2.53 ± 0.06	-	-	*1.46 ± 0.04*	*1.80 ± 0.05*	-	-
256	1.88 ± 0.05	2.50 ± 0.06	-	-	*1.46 ± 0.01*	*1.78 ± 0.04*	-	-

Table SI-I: The values of the critical exponents τ_1 and τ_2 and α_1 and α_2 for samples of different thicknesses l, estimated by fitting the expression Eq. (1) to the data shown in Fig. 4 in the main text.

1.2 Figure SI-1: Examples of fits with two slopes and cutoff

Some examples of these fits are shown in Fig. SI-1 (see also Fig.5 in the main text) and the corresponding parameters are shown.

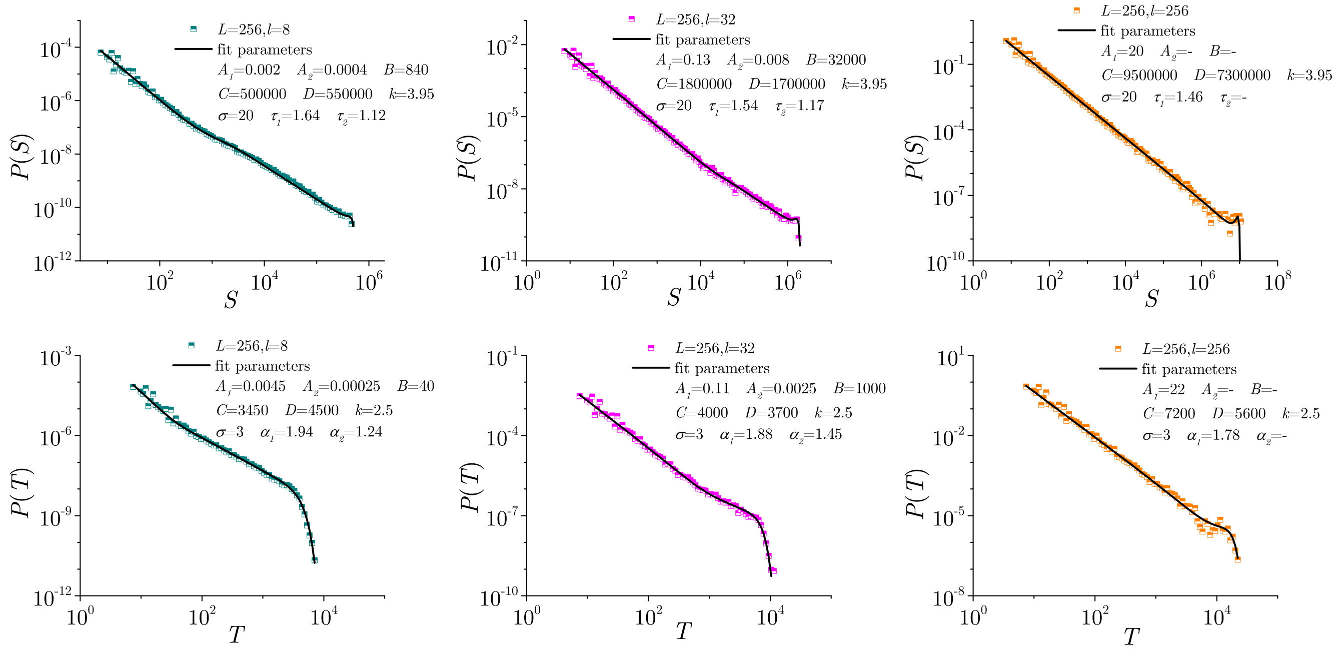

Figure SI-1: Illustration of fits according to Eq. (1-2) for three representative thicknesses for the distribution of avalanche size S and duration T in the HLC. Left: thin samples ($l = 8$); Middle: intermediate thickness ($l = 32$); Right: thick samples ($l = L = 256$). Corresponding parameters are listed in each panel. Parameters related to the second slope are not applicable in thick samples.

2 Comparisons with theoretical predictions

An analytical form of the distribution of the avalanche sizes with a bump, similar as in our simulations, has been derived by functional renormalization group theory for *the interface depinning* dynamics, see [1, 2] and other references in the main text. Correctly, the avalanche size distribution reads [1]

$$P(S) = \frac{\langle S\rangle}{2\sqrt{\pi}} S_m^{\tau-2} A S^{-\tau} \exp\left(C\sqrt{\frac{S}{S_m}} - \frac{B}{4}\left[\frac{S}{S_m}\right]^{\delta}\right) \tag{3}$$

where the exponents τ and δ as well as the parameters A, B, C are estimated from the ϵ-expansion. Precisely, from the one-loop expansion, these parameters are determined [1] (we are quoting only the case applicable to RF model):

$$\tau = 3/2 - \epsilon/12;\ \delta = 1 + \epsilon/18$$

while

$$A = 1 - 0.26835\epsilon/36;\ B = 1 + 2.28806\epsilon/9;\ C = \epsilon\sqrt{\pi}/9.$$

In the RG theory, the parameter $\epsilon \equiv d_c - d$, where $d_c = 4$ for the interface depinning model. Therefore, for comparisons of these theoretical curves with the data in 3-dimensional systems, we set $\epsilon = 1$, while $\epsilon = 2$ for the 2-dimensional case. Here, $< S >$ is the average size of the avalanche according to that distribution, while S_m is a fitting parameter [2]. Note that in this theoretical approach, no expression was given for the related distribution of avalanche duration.

2.1 Fits for the distribution of avalanche sizes

It should be stressed that only the avalanches in the central part of the hysteresis loop can have an extended domain wall, such that the interface dynamics can be dominant. By setting $\epsilon = 1$ in the above expressions, we attempt to fit our simulated data for the avalanche size distribution in HLC for large thickness, i.e., $l = L = 256$; the fit is shown in the top-right panel in Fig. SI-2 and Fig.5 in the main text. In this case, the RG theory provides a satisfactory fit, similar to the corresponding fit in Fig. SI-1 top-right, but with fewer parameters. The situation is much different in the case of finite thicknesses, for example, when $l = 8$ and $l = 32$, where the simulated distribution has two distinct slopes and a bump in the cut-off, see Fig. SI-2 left and middle. Here, the use of the expression of (3) with $\epsilon = 2$ to separately fit the *second part* of the simulated distribution is justified, where large 2-dimensional avalanches contribute. (For this purpose, we introduce additional fit parameter $\tilde{A} \equiv A\langle S\rangle$ and similarly for the separate fitting of the first part of the distribution, absorbing the actual value of $\langle S\rangle$.) In comparison with the corresponding fits in Fig. SI-1, we note that apart from fitted both slopes by the heuristic expression (1), the scaling exponent τ varies with the thickness. Moreover, the exponent $k = 3.95$ that fits the cut-off differs from the theoretical one 1/2. Apart from higher-order contributions to the expression (3), these differences call for a new RG theory for systems with finite thicknesses, which will also account for the scaling forms that involve additional scaling variable l/L [6].

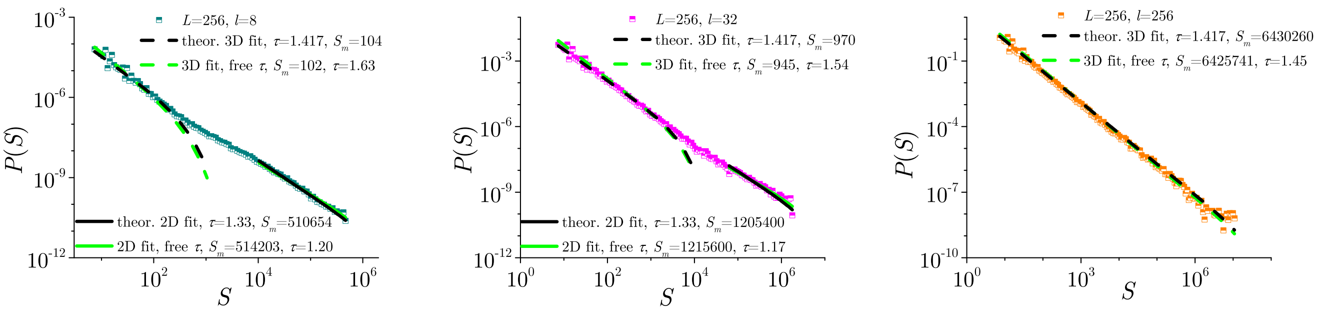

Figure SI-2: Representative fits of the simulated data in the HLC with the theoretical curve Eq. (3) and the corresponding parameters: For the data for thin sample $l = 8$ (left) and intermediate $l = 32$ thickness (middle), the second part of the distribution is fitted using $\epsilon = 2$, i.e., 2-dimensional system, while the first part has the slope of a 3-dimensional system. The data for thick sample $l = L = 256$ (right) are fitted using the parameters for $\epsilon = 1$, i.e., 3-dimensional system.

2.2 Shape of avalanches

The RG theory also predicts the asymmetric shape of the avalanches $n(t = xT) \sim [Tx(1-x)]^{\gamma-1} \times \exp(A_d(1/2 - x))$ with the exponential term, see [4] and the ϵ-expansion for the asymmetry parameter A_d as

$$A_d \approx -0.336(1 - d/d_c)\;, d = d_c - \epsilon\;. \qquad (4)$$

Notice that in the empirical expression [3] in Eq. (5) in the main paper, the asymmetric term represents the first order expansion of the above exponential expression with the asymmetry parameter $a \equiv A_d$. In [5], by performing an analysis of many samples with different thicknesses and the avalanche duration T up to 2048, the corresponding values of the parameter a are found to be negative and dependent on the actual thickness in agreement with the RG predictions (4), see Table SI-II for details. It is interesting to notice that, by taking $d_c = 4$, the expression (4) gives the value a=-0.084 for $d = 3$, while a=-0.168 for $d = 2$; the latter value is close to the numerical one for large avalanche durations in the thin sample.

Table SI-II: Values of the avalanche asymmetry parameter a and the corresponding γ obtained from fitting the avalanche shapes for the avalanche duration $T \in [64, 2048]$ and thickness $l \in [1, 512]$, data from [5]

(γ)	$T = 64$	$T = 128$	$T = 256$	$T = 512$	$T = 1024$	$T = 2048$
$l = 1$	1.42	1.44	1.45	1.47	1.49	$-$
$l = 2$	1.47	1.48	1.48	1.49	1.48	1.45
$l = 4$	1.50	1.50	1.48	1.49	1.48	1.48
$l = 8$	1.50	1.55	1.54	1.50	1.48	1.48
$l = 16$	1.51	1.54	1.58	1.59	1.53	1.51
$l = 32$	1.53	1.55	1.57	1.58	1.61	1.60
$l = 64$	1.54	1.57	1.58	1.60	1.59	1.67
$l = 128$	1.54	1.58	1.59	1.59	1.62	1.59
$l = 256$	1.54	1.58	1.70	1.62	1.61	1.65
$l = 512$	1.54	1.58	1.60	1.61	1.62	1.63

(a)	$T = 64$	$T = 128$	$T = 256$	$T = 512$	$T = 1024$	$T = 2048$
$l = 1$	-0.351	-0.292	-0.241	-0.189	-0.173	$-$
$l = 2$	-0.334	-0.276	-0.221	-0.185	-0.156	-0.110
$l = 4$	-0.418	-0.346	-0.277	-0.228	-0.196	-0.161
$l = 8$	-0.264	-0.344	-0.322	-0.270	-0.217	-0.173
$l = 16$	-0.217	-0.199	-0.244	-0.284	-0.243	-0.199
$l = 32$	-0.229	-0.198	-0.194	-0.183	-0.238	-0.256
$l = 64$	-0.236	-0.198	-0.184	-0.165	-0.171	-0.205
$l = 128$	-0.236	-0.199	-0.179	-0.177	-0.154	-0.147
$l = 256$	-0.236	-0.202	-0.175	-0.160	-0.177	-0.167
$l = 512$	-0.236	-0.196	-0.171	-0.148	-0.183	-0.166

3 Distributions away from the critical line

To add to the discussion in the main paper, here we present the simulation results for the situations that typically occur in empirical investigations, that is away from the critical line.

3.1 Fixed disorder—varied thickness

This situation often occurs in experiments; it represents a horizontal cut through the phase diagram, cf. Fig. SI-5.

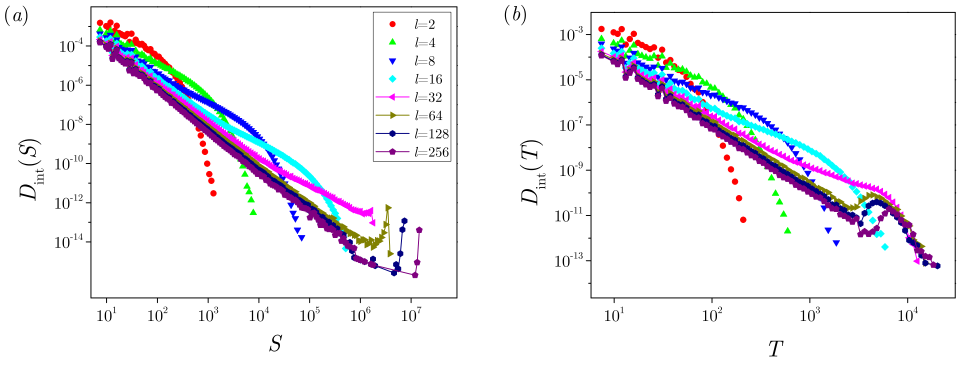

Figure SI-3: Integrated distributions of avalanche size S, panel (a), and of avalanche duration T, panel (b), for the systems with same disorder $R = R_c^{\rm eff}(l = 32)$ and base size $L = 256$, but with various thicknesses, ranging from $l = 2$ to $l = 256$. The distributions are obtained after averaging over different configurations of the random magnetic field (500 for $l = 256$, and increasing up to 60000 for $l = 2$).

3.2 Fixed thickness—varied disorder

This situation represents a vertical cut through the disorder–thickness phase diagram. In the laboratory experiments, it assumes samples of a different composition, as in the case of thin films, cf. . SI-5. Theoretically, below the critical disorder line, the interface depinning occurs, and the distributions dominate with the spanning avalanches. Whereas, above the critical line, the cut-offs are limiting the power-law distributions, with a systematically decreasing cut-off length. The nature of the accompanying Barkhausen noise is changed accordingly, as shown in Fig.8 in the paper.

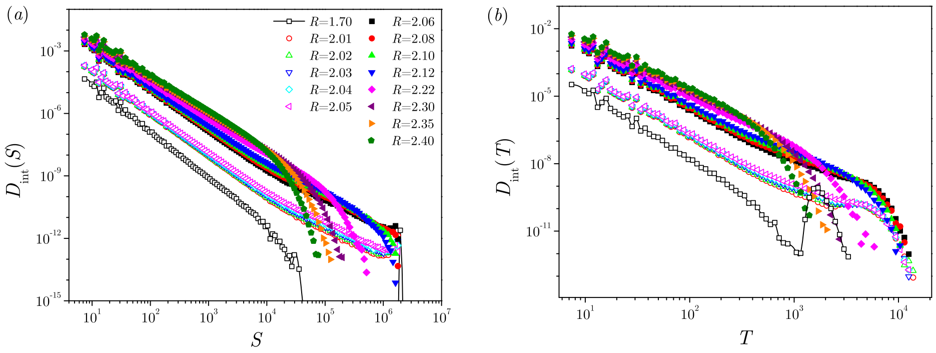

Figure SI-4: Integrated distributions of avalanche size S, panel (a), and of avalanche duration T, panel (b), for the systems with fixed thickness $l = 32$ and base size $L = 256$, but with various disorder R in the range above and below the critical $R_c^{\rm eff}(l = 32)$. The distributions are obtained after averaging over 4000 different configurations of the random magnetic fields.

4 Comparisons with experimental results

In Fig. SI-5, we plot the disorder–thickness phase diagram using the relative scales (see the figure caption), that enable to plot several experimental results from the literature relative to the studied critical disorder line.

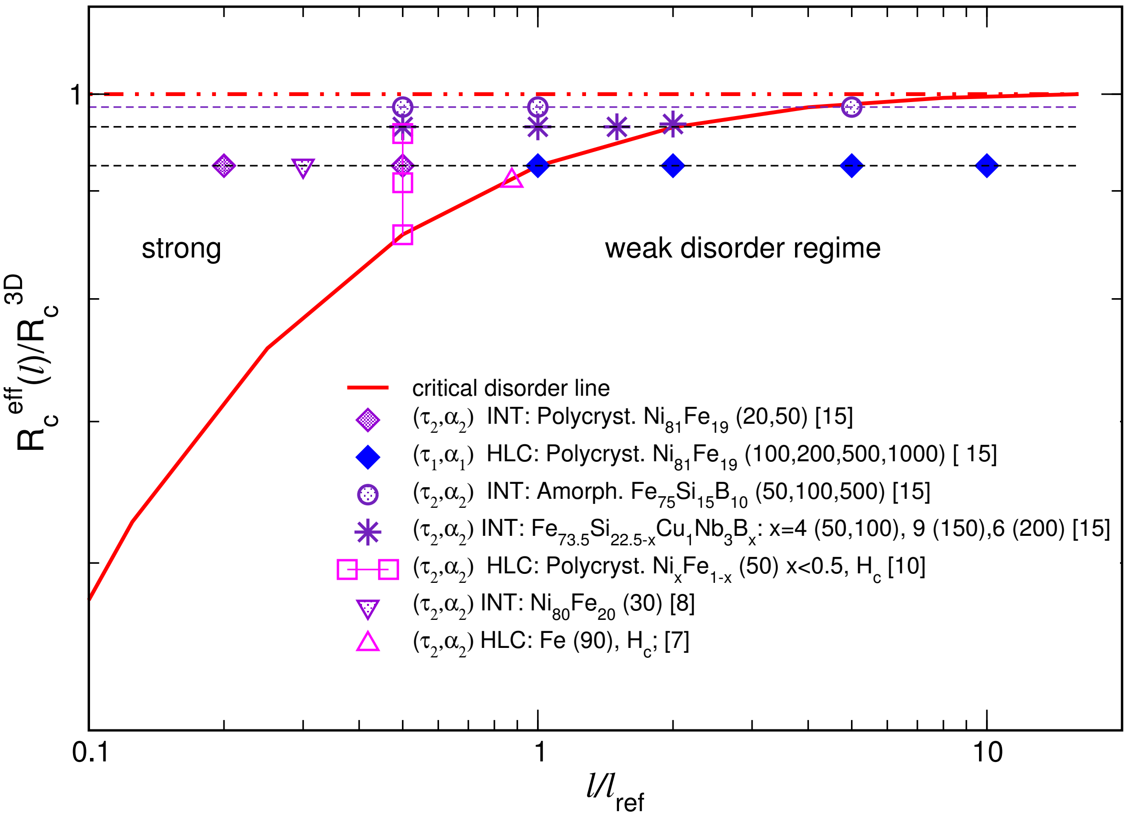

Figure SI-5: Position of the critical disorder line $R_c^{\rm eff}(l,512)$ normalised with the critical disorder in bulk sample $R_{c,3D}$ plotted against the relative thickness scale l/l_{ref}, and the comparison with the available experimental results in Refs. [7,8,10,11,15]. The reference thickness l_{ref} was identified as the thickness where the avalanche exponents change in agreement with the predicted theoretical values; it corresponds to 100 nm thickness in the polycrystalline sample $\rm Ni_xFe_{1-x}$ from the most complete measurements of different thicknesses in Ref. [15], where $x = 81$, and a horizontal line is set at the equivalent theoretical critical disorder. Then all other thicknesses from different measurements are placed relative to that point. Different measurements obtained in the alloy of the same composition belong to the same line, while the points representing a varied composition at the fixed thickness belong to a vertical line, where we can not assess the actual disorder but only plot the theoretical lower and upper limits. Similar reasoning are applied to assess relative disorder line for other samples, in particular, amorphous $\rm Fe_{75}Si_{15}B_{10}$ and $\rm Fe_{73.5}Si_{22.5-x}Cu_1Nb_3B_x$; here, the exponents measured in Ref. [15] systematically remain in the range left/above the theoretical critical line, suggesting a stronger disorder in these amorphous alloys. In the legend, we indicate the theoretical exponents that correspond (within error bars) to the experimental values in the respective materials: (τ_2, α_2) referring to the theoretical second slope observable in thin materials, while (τ_1, α_1) are the exponents of the first slope, which dominates in the distributions computed for the thicker samples, see Methods in the main text for the exact definition. The abbreviation "HLC" indicate that these exponents are observed by considering strictly central segments of the hysteresis loop and "INT" indicates integration over an extended segment of the loop (or loop branch). H_c in the legend indicates that these experiments were done by imposing the field close to the coercive value (the corresponding symbols are empty), in contrast to field sweeping with a given rate as it is standardly done in other experiments. The corresponding range of the theoretical exponents are highlighted in the Table SI-I as (τ_2, α_2) INT—bold, (τ_1, α_1) HLC—italic, and (τ_2, α_2) HLC—italic-bold fonts.

Bibliography

[1] Rosso, A., Le Doussal, P. & Wiese, K.J. Avalanche-size distribution at the depinning transition: A numerical test of the theory. *Phys. Review B*, 80:144204, 2009.

[2] Le Doussal, P., Middleton, A.A. & Wiese, K.J. Statistics of static avalanches in random pinning landscape. *Phys. Rev. E* 79:050101(R), 2009.

[3] Laurson, L., Illa, X., Santucci, S., Tallakstad, K.T., Måløy, K.J. & Alava, M.J. Evolution of the average avalanche shape with the universality class. *Nature Communications*, 4:2927, 2013.

[4] Dobrinevski, A., Le Doussal, P. & Wiese, K.J. Avalanche shapes and exponents beyond mean-field theory. *EPL*, 108:66002, 2014.

[5] Mijatović, S. Crossover from three-dimensional to two-dimensional systems and impact of number of neighbours on critical behaviour of the nonequilibrium zero-temperature random field Ising model. Ph.D. Thesis (in Serbian), UDC 533.9(043.3), Faculty of Physics, University of Belgrade, 2019.

[6] Spasojević, D., Mijatović, S., Navas-Portella, V. & Vives, E. Crossover from three-dimensional to two-dimensional systems in the nonequilibrium zero-temperature random-field Ising model. *Phys. Rev. E*, 97:012109, 2018.
